# A patient-specific multi-modality abdominal aortic aneurysm imaging phantom

**DOI:** 10.1007/s11548-022-02612-4

**Published:** 2022-04-10

**Authors:** Callum D. Little, Eleanor C. Mackle, Efthymios Maneas, Debra Chong, Daniil Nikitichev, Jason Constantinou, Janice Tsui, George Hamilton, Roby D. Rakhit, Tara M. Mastracci, Adrien E. Desjardins

**Affiliations:** 1 Wellcome Trust-EPSRC Centre for Interventional and Surgical Sciences, London, W1W 7TS UK; 2 Department of Medical Physics and Bioengineering, University College London, London, WC1E 6BT UK; 3 Department of Cardiology, Royal Free Hospital, London, NW3 2QG UK; 4 Department of Vascular Surgery, Royal Free Hospital, London, NW3 2QG UK; 5 Division of Surgery and Interventional Science, University College London, London, W1W 7TY UK

**Keywords:** Ultrasound, Imaging phantoms, Tissue-mimicking material, Vascular, Abdominal aortic aneurysm

## Abstract

**Purpose:**

Multimodality imaging of the vascular system is a rapidly growing area of innovation and research, which is increasing with awareness of the dangers of ionizing radiation. Phantom models that are applicable across multiple imaging modalities facilitate testing and comparisons in pre-clinical studies of new devices. Additionally, phantom models are of benefit to surgical trainees for gaining experience with new techniques. We propose a temperature-stable, high-fidelity method for creating complex abdominal aortic aneurysm phantoms that are compatible with both radiation-based, and ultrasound-based imaging modalities, using low cost materials.

**Methods:**

Volumetric CT data of an abdominal aortic aneurysm were acquired. Regions of interest were segmented to form a model compatible with 3D printing. The novel phantom fabrication method comprised a hybrid approach of using 3D printing of water-soluble materials to create wall-less, patient-derived vascular structures embedded within tailored tissue-mimicking materials to create realistic surrounding tissues. A non-soluble 3-D printed spine was included to provide a radiological landmark.

**Results:**

The phantom was found to provide realistic appearances with intravascular ultrasound, computed tomography and transcutaneous ultrasound. Furthermore, the utility of this phantom as a training model was demonstrated during a simulated endovascular aneurysm repair procedure with image fusion.

**Conclusion:**

With the hybrid fabrication method demonstrated here, complex multimodality imaging patient-derived vascular phantoms can be successfully fabricated. These have potential roles in the benchtop development of emerging imaging technologies, refinement of novel minimally invasive surgical techniques and as clinical training tools.

## Introduction

Abdominal aortic aneurysms are highly complex three dimensional anatomical structures of particular interest within the field of Vascular Surgery, due to the risk of a potential aneurysmal rupture [1]. Endovascular intervention is often the preferred treatment option over an open surgical approach due to decreased patient recovery time and risk of wound complications [2].

Traditionally, surgical training has been undertaken on human and animal cadavers, before progression to supervised participation on patients. Endovascular aortic repair (EVAR) procedures are particularly challenging to learn due to the need for radiological familiarisation and the requirement for complex psychomotor skills to manipulate the endovascular devices. Furthermore, shortened post-graduate training programmes and increasing clinical commitments can limit the opportunities that trainees have to gain experience and competency with these procedures [3].

Phantom models are powerful tools for trainee surgeons learning to perform these procedures. They can provide both a realistic representation of patient anatomy and an opportunity to gain familiarity with surgical equipment and techniques, without risking harm to a patient. Simulation training on phantom models has been shown to result in decreased fluoroscopy and procedural times [4, 5] as well as overall volume of contrast used [6], as compared to traditional teaching methods. Additionally, patient-specific phantom models can be used in the preoperative planning of interventions [7, 8]. Phantom models can also provide opportunity for benchtop characterization of emerging procedural devices and techniques, such as in-situ fenestration of EVAR [9], prior to in-vivo testing.

Current simulation tools include both virtual reality (VR) platforms and physical phantoms. Although VR training platforms have shown promising outcomes in EVAR, with decreased procedural time and post-procedure endoleaks observed [10], physical phantoms are also important tools for surgeons seeking to develop the complex motor skills required for EVAR procedures [11]. However, most commercially available models have many limitations. Currently, phantoms that are low-cost often tend to have a low level of anatomical accuracy and are not typically patient specific, which limits their use for surgical planning or in-depth training. Others that are more anatomically realistic tend to be very expensive, and even those may only be compatible with one imaging modality. Many methods for creating AAA models in the literature include the use of high cost or difficult-to-source materials and techniques, such as stereolithography with UV-curing resins [12] 13, so they cannot readily be reproduced for clinical training. Additionally, most current phantoms include only the vessel structures [14] without surrounding tissues, so that their realism and use with multimodality imaging are limited.

Image fusion (IF) is a technique where data from one imaging modality are combined with another to produce an overlay in real time. Within the field of vascular surgery, it has an increasing role in the endovascular treatment of complex aortic aneurysms, and therefore, it is important that EVAR training phantoms are designed to be compatible with IF. Using IF, preoperative CT data can be overlaid onto intra-procedural fluoroscopic images, to facilitate fenestrated stent deployment [9, 15]. This requires fixed radiological landmarks, such as the spine, to be visible on both of the imaging modalities.

Here, we present a novel technique based on 3D printing for creating anatomically accurate AAA phantoms, which can be used in the simulation of EVAR procedures. The methods and materials resulted in a model that is compatible with computerized tomography (CT) and ultrasound (US), and with IF. The geometry of the vascular structures was derived from patient data. The utility of this phantom is then demonstrated during a simulated EVAR procedure.

## Methods

### Phantom fabrication

**Fig. 1:**
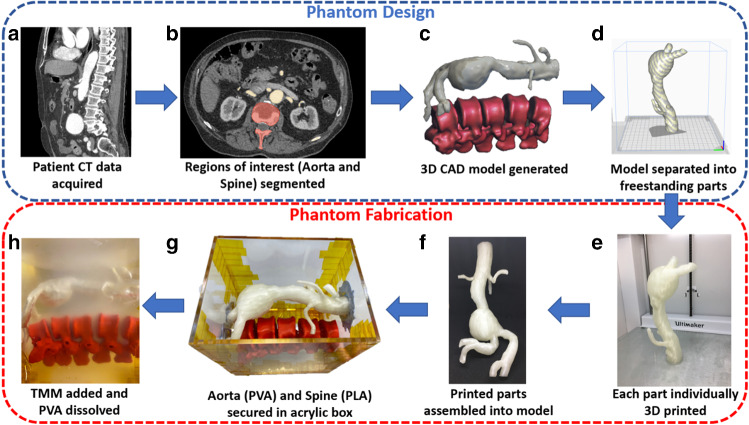
Phantom model fabrication. (**a**) Volumetric data are acquired from a patient CT scan and (**b**) regions of interest from this data-set are manually segmented; (**c**) these segments are then exported as 3D computer aided design models and the mesh is refined to create an STL file compatible with 3D printers; (**d**) the models are orientated to negate the requirement for support material; (**e**) large overhanging structures are separated and printed individually using PVA before (**f**) assembly into the completed model; (**g**) the printed Aorta and Spine are secured in an acrylic box with both ends of the PVA Aorta exposed; (**h**) tissue mimicking material is integrated into the box and the inner PVA Aorta is dissolved out using water

There are several key steps in the fabrication method presented, which are illustrated in Fig. 1. Firstly, patient CT data are acquired allowing segmentation of areas of interest. Next, the segmentations are converted into models for 3D printing; the vasculature is then printed in a water-soluble material, whilst bone structures are printed in a non-water-soluble material. Finally, the phantom is constructed by pouring a tissue-mimicking material (TMM), which has the radiological appearance of human soft tissue, over the 3D printed model. Once the TMM has set, the phantom is submerged in water to dissolve out the vascular structures, producing a hollow, or “wall-less” phantom.

#### Model generation

For both models, volumetric patient-specific data were obtained from arterial phase contrast CT acquisitions (Fig. 1a). The data set was segmented manually, using an open source medical image analysis software platform (3D Slicer), according to vascular regions of interest (Fig. 1b). A 3D model of the segmented data was exported to Meshmixer (Autodesk Inc, San Rafael, CA, USA) to allow for smoothing and refinement (Fig. 1c), before generation of an STL-file compatible with 3D extrusion printing software. Unsupported structures with an overhead angle to the base plate of greater than 70˚ were segmented into separate models, to prevent the requirement for support material during the printing process, before later amalgamation (Fig. 1d). The wall thickness of the model was 1 mm with a layer height of 0.15 mm.

#### 3D printing

The models were printed using a dual extrusion, commercially available 3D printer (Ultimaker S5, Ultimaker B.V., Utrecht, Netherlands) (Fig. 1e). The material used for printing was Poly(vinyl alcohol) (PVA) (RS components Ltd, Northants, UK) which is a water soluble, non-toxic filament commonly used as a support material for 3D printing. Once printed, the separate segments were combined together to form the final model using PVA glue (Elmer’s Products Inc, NC, USA), a water-soluble adhesive (Fig. 1f). The aorta model was secured in a custom laser-cut acrylic open-topped box, with either end of the main vascular structure exposed (Fig. 1g). An additional insoluble spine, 3D printed using Polylactic acid (PLA), was included within the model. This provided an anatomical landmark, allowing for image fusion (IF) guidance techniques and facilitating benchtop endovascular procedures.

#### Tissue-mimicking material

Gel-wax has recently emerged as a promising TMM and has been used to create heterogenous anatomical ultrasound tissue phantoms [16, 17]. It is an insoluble, mineral-oil based material that is solid at room temperature. It has a low melting point of 70˚C, above which it becomes highly mobile. Ultrasound properties can be tuned through the addition of glass spheres. Gel-wax offers several advantages compared to other TMMs such as PVA or silicone. Firstly, it is both mechanically and thermally stable; secondly, it has a low viscosity, facilitating integration into phantom models; lastly it is insoluble in water which facilitates generation of wall-less phantom models when using a water-soluble inner model.

The gel-wax (Mindsets UK, Essex, UK) was heated to a temperature of 150˚C for a period of 4 h to ensure uniform consistency. Air removal was facilitated using a vacuum chamber before glass microspheres were added in a concentration of 0.5% for acoustic scattering. The mixture was sonicated for 5 min, to guarantee uniform particle distribution and achieve acoustic homogeneity. The mixture was introduced into the acrylic open-topped box, allowing the gel-wax to surround the PVA aorta and PLA spine models, cooling into a solid state. Finally, the box was immersed in de-ionised water for 24 h. The exposed ends of the main vascular structure provided an entry point for the water resulting in the dissolution of the PVA aorta, leaving behind a negative, wall-less vascular structure, surrounded by tissue-mimicking material (Fig. 1h).

### Imaging

#### Transcutaneous ultrasound

Transcutaneous ultrasound imaging was obtained using a commercially available clinical system (HS40, Samsung Healthcare, Seoul, South Korea) with a curved array transducer probe (C2-8) operating with a frequency range of 2–6 MHz. The phantom was submerged in water to remove acoustic shadowing from air.

#### Intravascular ultrasound

Intravascular ultrasound was performed using an intracoronary imaging system (Opticross, Boston Scientific, Marlborough, MA, USA) operating with a transducer frequency of 40 MHz. A 0.018-in. diameter coronary guidewire (Hi-Torque Balance Middleweight, Abbott Cardiovascular, MN, USA) was passed through the water-filled phantom and fixed at either end. The imaging catheter was then loaded onto the coronary guidewire and positioned in the region of an iliac vessel. Images were acquired with an automated pullback speed of 1 mm/s.

#### Computerised tomography

CT acquisition was acquired using a 320-slice scanner (Aquillion One, Canon Medical Systems Corporation, Tochigi, Japan), with a CT angiography protocol (thickness 0.5 mm; 100 kV; rotation time 0.5 s; mA R 388). To provide realistic contrast enhancement for the vascular structures, the phantom was submerged in a mixture of water and Iodixanol (Visipaque, GE Healthcare, Chicago, IL, USA), a water soluble, isosmolar, non-ionic, iodinated radiographic contrast agent.

## Results

**Fig. 2:**
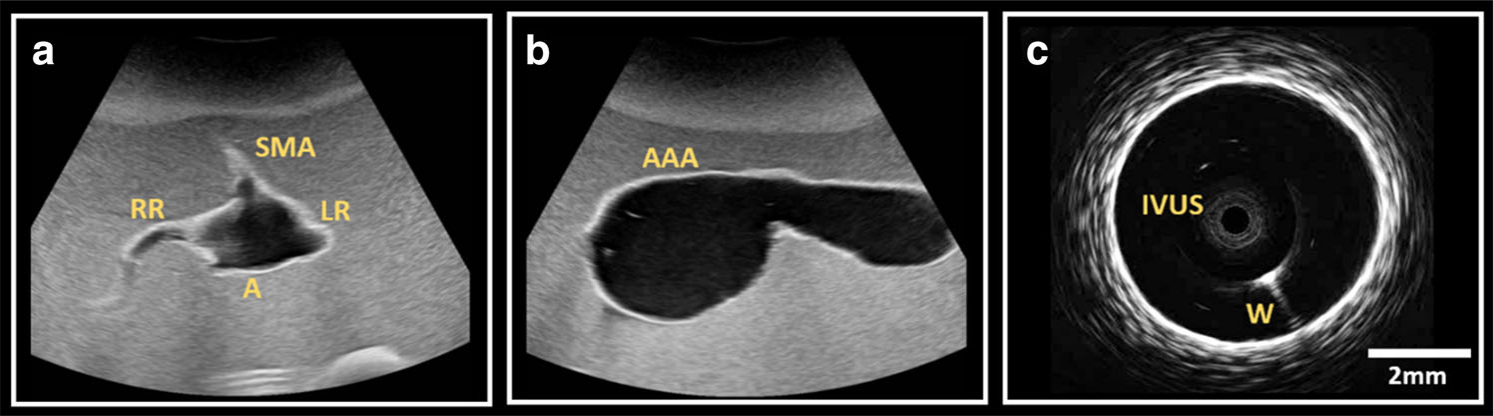
Ultrasound imaging of the phantom model. (**a**) External ultrasound demonstrating an axial view of the Aorta (**A**) surrounded by an ultrasonically homogenous tissue mimicking material. Left (LR)/ Right (RR) Renal arteries and the Superior Mesenteric Artery (SMA) origins are visible. (**b**) A longitudinal view demonstrating the Abdominal Aortic Aneurysm sac (AAA). (**c**) Intravascular ultrasound with the imaging catheter (IVUS) located centrally within the lumen of the vessel. W represents acoustic artefact created by the coronary guidewire used to insert the imaging probe

With transcutaneous ultrasound imaging various anatomical areas of interest were clearly delineated (Fig. 2a). The transcutaneous axial view (Fig. 2a) demonstrates the main aorta (A), renal (LR/RR), and superior mesenteric (SMA) arteries, visible as hypoechoic areas. The TMM appears as a homogenous hyperechoic medium surrounding these structures. In the longitudinal view (Fig. 2b) the abdominal aortic aneurysm sac (AAA) and neck are visible. Intravascular ultrasound of the phantom (Fig. 2c) demonstrates an echo-bright cylindrical structure consistent with the appearance of vascular endothelium, with underlying low attenuation material consistent with the appearance of adventitia.

**Fig. 3:**
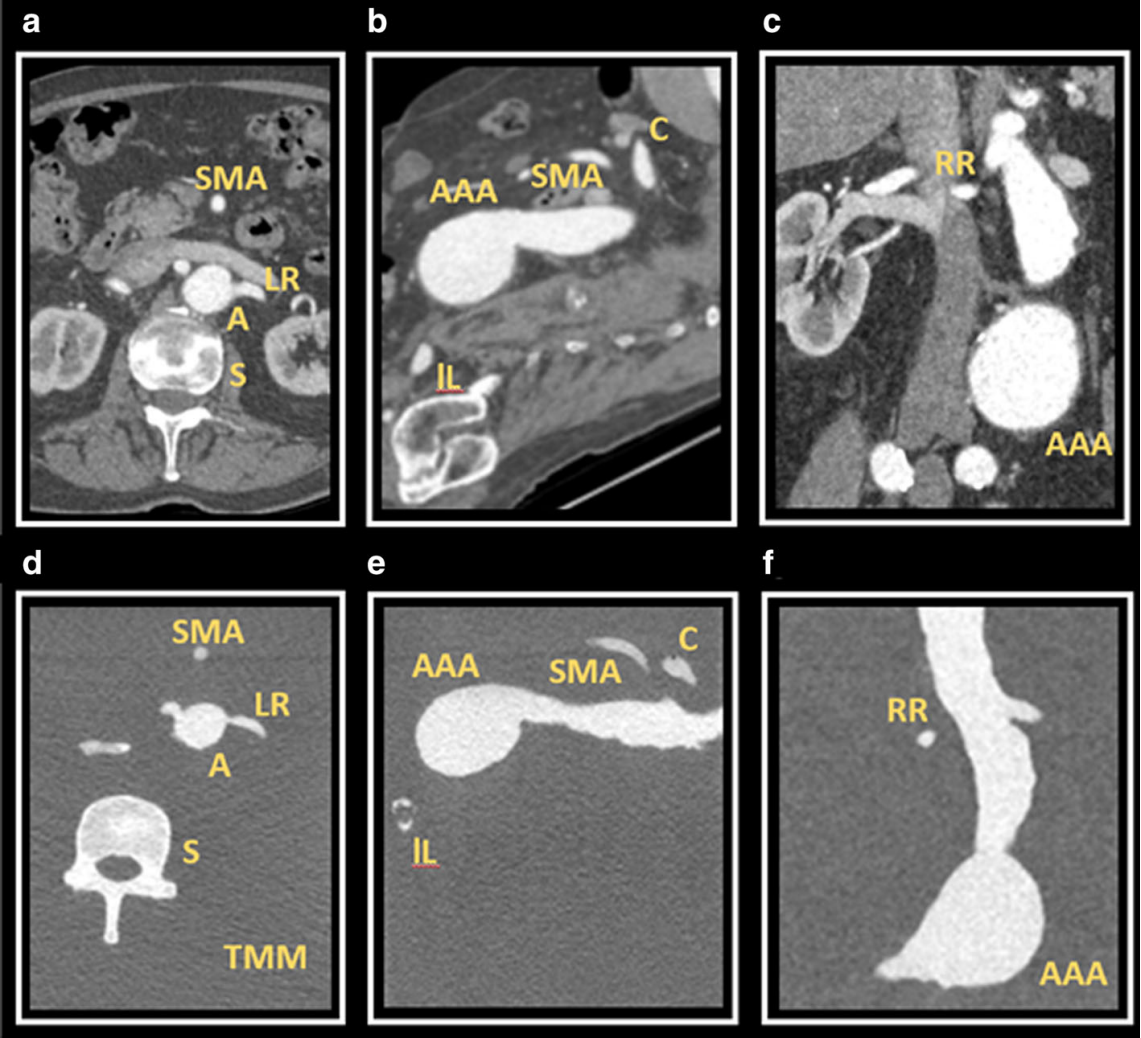
Comparison of CT acquisitions from both the patient with an abdominal aortic aneurysm and the patient-derived phantom model. (**a**) Axial view of patient CT at the level of the renal artery bifurcation with the left renal artery (LR), spine (S) and aorta (A) demonstrated; (**b**) Corresponding axial view from the phantom model CT demonstrating the same structures surrounded by tissue mimicking material (TMM); (**c**) Sagittal view of patient CT demonstrating abdominal aortic aneurysm (AAA) sac, coeliac artery (C), superior mesenteric artery (SMA) and iliac artery (IL); (**d**) Corresponding sagittal view from the phantom model CT; (**e**) Coronal view of patient CT demonstrating the abdominal aortic aneurysm (AAA) and right renal artery (RR); f) Corresponding coronal slice of phantom model CT

The CT acquisition of the phantom was compared to the original patient CT dataset from which the phantom was derived (Fig. 3). Three different radiological planes, at similar slices, are demonstrated; axial **(**Fig. 3a, b), Sagittal (Fig. 3c, d) and coronal (Fig. 3e, f). In both acquisition sets, there is contrast clearly visible within the vascular structures, allowing for delimitation from surrounding material. The aneurysmal sac of the complex abdominal aortic aneurysm (AAA) is seen in two orthogonal views (Fig. 3d, f), with orientation of visceral side branches, such as Iliacs (IL), Renals (RR/LR), Superior Mesenteric (SMA) and Coeliac (C), preserved. The PLA spine (S) included in the phantom model is visible as a radiopaque structure (Fig. 3b) and corresponds with the appearance of the spine from the patient’s CT scan (Fig. 3a).

**Fig. 4:**
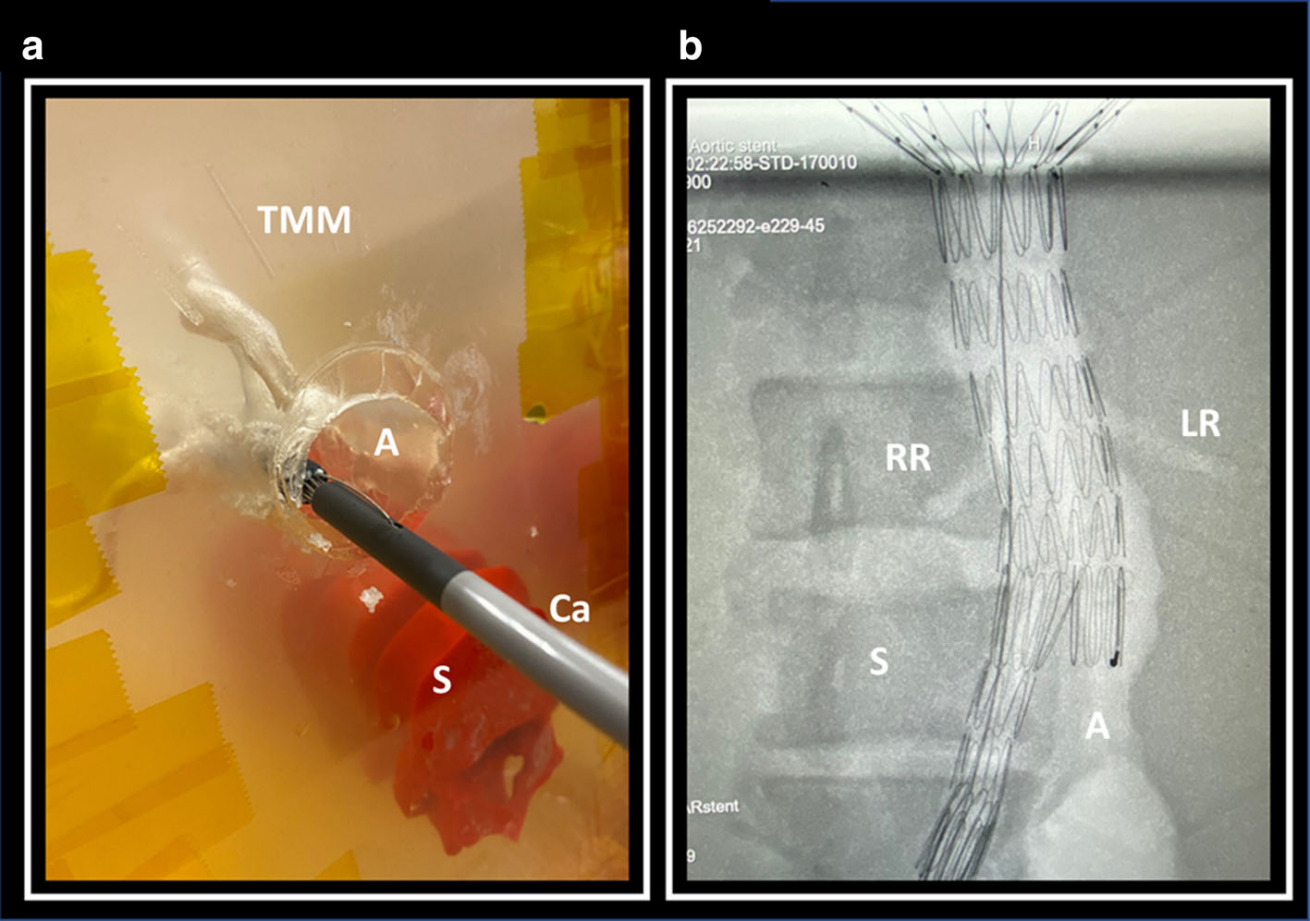
Simulated EVAR procedure. (**a**) Insertion of catheter (Ca) into the aorta (A) during the simulated EVAR procedure with the spine (S) visible within the tissue mimicking material (TMM); (**b**) anterior–posterior fluoroscopic view of the phantom model post EVAR stent deployment. The spine (S) is visible as a hyperdense structure with the left renal artery (LR), right renal artery (RR) and aorta (A) visible as hypodense structures

The phantom model was utilised successfully as part of an EVAR training simulation. A trainee with no prior experience of EVAR deployed a AAA endovascular bifurcated stent (Zenith Flex, Cook Medical, Bloomington, IL, USA) under the guidance of a Consultant Vascular Surgeon (Fig. 4a). Access to the aorta was obtained through the right iliac. Fluoroscopy was used to confirm positioning of the device (Fig. 4b).

## Discussion

The method of fabrication that we have presented here can be used to create a high-fidelity patient specific vascular phantom of an AAA, compatible with both US and CT imaging. The novelty of this study stems from several factors. Firstly, the use of 3D printed dissolvable vascular models embedded within TMM. Secondly, we have demonstrated the phantom’s utility as a training tool during simulation of an EVAR procedure. The materials used in the fabrication process are inexpensive, stable at room temperature and have no specific limitations on shelf-life. The total material cost of the phantom was less than €100. Similar phantoms with material costs of up to €2000 are quoted in the literature as reaching [13]. The use of 3D printed dissolvable models allows for complex geometries to be generated, which not achievable using current techniques. The inclusion of radiopaque landmarks within the model ensures compatibility with image fusion guidance techniques, which help facilitate detection of visceral side-branches during complex EVAR procedures [15]. A further advantage of this fabrication method is that the process of dissolving the printed structures out of the model leaves behind a hollow, wall-less vasculature. This then has a realistic ultrasound appearance, and does not have the highly reflective signals that are present using traditional silicone tubing or resin-based imaging phantoms.

Whilst the potential utility of the fabricated AAA phantom model has been demonstrated in the simulation of an EVAR procedure, further evaluation in the form of surgeon feedback and objective outcome data is required to determine the efficacy of this as a training tool. It is, however, envisaged that other complex vascular structures, such as coronary or cerebral vessels, can be recreated using the same method. This may allow fabrication of suitable phantoms for simulation of procedures relevant to trainee Interventional Cardiologists or Radiologists, provided accurate luminal casts can be made of small vessels. In addition to compatibility with transcutaneous ultrasound, the phantom model produces realistic imaging when IVUS is used. IVUS has a role in EVAR procedures allowing accurate sizing of vessels and reducing contrast volume [18]. IVUS is also frequently used to guide coronary and peripheral vascular angioplasty and therefore compatibility with this imaging modality broadens the applicability of this phantom. Compatibility with both IVUS and transcutaneous US could allow for EVAR procedures to be performed with reduced ionising radiation. For instance, emerging ultrasonic tracking technologies with fibre optic receivers integrated into devices [19] could be used to accurately position the stent graft; likewise, emerging rotational intravascular technologies could be used to align fenestrations with side branches [20].

The smallest vessel size fabricated in this model was the right renal artery (ca. 5 mm diameter); however depending on the resolution of the 3D printer system used, it may be possible to include vessels as small as 1 mm in diameter [21]. This would allow reproduction of complex arteriole vascular beds, such as those seen with arterio-venous malformations [22] if a good luminal model could be created. Indeed, the utility of dissolvable structures is not limited to recreation of vascular structures but can also potentially be used to simulate other hollow branching systems such as lymphatics, ureters or the biliary tree. Surrounding these structures with tissue mimicking material, cast in organ-specific shapes, could allow for realistic imaging phantoms for visceral organs such as the liver, kidney or prostate. The TMM used was specific to ultrasound imaging, however inclusion of alternative materials with differing imaging contrast could potentially allow for compatibility with further imaging modalities such as magnetic resonance imaging (MRI). Furthermore, the elastic properties of the tissue mimicking material used in this study can be altered by the addition of paraffin oil [17], allowing further tailoring of vascular phantoms for use with flow simulations in a wide range of clinical contexts [23, 24].

## Conclusions

Through a hybrid approach including 3D printing vascular structures in water-soluble material, and integration of tissue mimicking materials, anatomically complex multimodality patient-specific imaging phantoms can be fabricated, which are of use in simulation training of EVAR.
